# Melatonin activates Parkin translocation and rescues the impaired mitophagy activity of diabetic cardiomyopathy through Mst1 inhibition

**DOI:** 10.1111/jcmm.13802

**Published:** 2018-07-31

**Authors:** Shanjie Wang, Zhijing Zhao, Xinyu Feng, Zheng Cheng, Zhenyu Xiong, Tingting Wang, Jie Lin, Mingming Zhang, Jianqiang Hu, Yanhong Fan, Russel J. Reiter, Haichang Wang, Dongdong Sun

**Affiliations:** ^1^ Department of Cardiology Xijing Hospital Fourth Military Medical University Xi'an China; ^2^ Department of Cardiology Tangdu Hospital Fourth Military Medical University Xi'an China; ^3^ Department of Cellular and Structural Biology UT Health Science Center San Antonio Texas

**Keywords:** diabetic cardiomyopathy, mammalian Ste20‐like kinase 1, Mst1, melatonin, mitochondrion, mitophagy, parkin

## Abstract

Mitophagy eliminates dysfunctional mitochondria and thus plays a cardinal role in diabetic cardiomyopathy (DCM). We observed the favourable effects of melatonin on cardiomyocyte mitophagy in mice with DCM and elucidated their underlying mechanisms. Electron microscopy and flow cytometric analysis revealed that melatonin reduced the number of impaired mitochondria in the diabetic heart. Other than decreasing mitochondrial biogenesis, melatonin increased the clearance of dysfunctional mitochondria in mice with DCM. Melatonin increased LC3 II expression as well as the colocalization of mitochondria and lysosomes in HG‐treated cardiomyocytes and the number of typical autophagosomes engulfing mitochondria in the DCM heart. These results indicated that melatonin promoted mitophagy. When probing the mechanism, increased Parkin translocation to the mitochondria may be responsible for the up‐regulated mitophagy exerted by melatonin. Parkin knockout counteracted the beneficial effects of melatonin on the cardiac mitochondrial morphology and bioenergetic disorders, thus abolishing the substantial effects of melatonin on cardiac remodelling with DCM. Furthermore, melatonin inhibited Mammalian sterile 20‐like kinase 1 (Mst1) phosphorylation, thus enhancing Parkin‐mediated mitophagy, which contributed to mitochondrial quality control. In summary, this study confirms that melatonin rescues the impaired mitophagy activity of DCM. The underlying mechanism may be attributed to activation of Parkin translocation via inhibition of Mst1.

## 1 INTRODUCTION

The global prevalence of diabetes is estimated to affect 439 million adults (aged 20‐79 years) by 2030. There will be a 69% increase in the number of diabetic patients in developing countries between 2010 and 2030.^1^ Diabetic cardiomyopathy accounts for a significant increase of mortality in diabetic patients.^2^ However, current treatment strategies for cardiomyopathy in diabetic patients are the same as in non‐diabetic patients and do not address the underlying causes of cardiac dysfunction.^3^, ^4^

Mitochondria are organelles that produce the energy that is required to drive the endergonic reactions of cell life and are part of the signalling process.^5^ The heart is rich in mitochondria to maintain normal contractile function due to the high energy demands of this organ as well as other tissues.^6^, ^7^, ^8^ Cardiac mitochondria play a critical role and act as a power plant for cardiomyocytes through oxidative phosphorylation.^9^, ^10^, ^11^ Increasing evidence suggests that the cardiovascular complications of diabetes converge on mitochondria as an epicentre for cardiomyocyte damage.^12^ Cardiomyocytes are particularly susceptible to diabetic insults caused by dysfunctional mitochondria.^13^, ^14^ Mitochondria that are damaged during diabetic stress can produce reactive oxygen species (ROS) and release death‐inducing factors, thus augmenting cardiomyocyte injury.^14^, ^15^, ^16^

Effective clearance of these damaged mitochondria may prevent cardiomyocyte damage induced by diabetes and limit cardiac dysfunction.^13^, ^17^ Autophagy is the primary mechanism for mitochondrial quality control and removes whole mitochondria. In contrast to non‐selective autophagy, mitophagy is a more specific mechanism that maintains a healthy mitochondrial population.^18^, ^19^, ^20^ During mitophagy, damaged mitochondria are selectively sequestered by phagophores, encapsulated and subsequently fused with lysosomes to recycle their essential components.^20^ Mitophagy ensures that cardiomyocytes maintain a functional network of mitochondria and provides strong protection against diabetic insults. Mitophagy can be regulated by several pathways during mitochondrial quality control.^13^, ^20^, ^21^ Amongst these, the PINK1/PARKIN pathway is well‐studied and is involved in the labelling of damaged mitochondria for mitophagy degradation.^22^ Thus, a better understanding of whether PINK1/PARKIN‐ mediated mitophagy participates in the pathogenesis of diabetic cardiomyopathy is still needed as well as whether they can be targeted therapeutically.

Melatonin (N‐acetyl‐5‐methoxytryptamine) is predicted to have evolved an estimated 3.0‐2.5 billion years ago.^23^, ^24^ The chemical structure of melatonin has remained very stable for billions of years, and its structure is identical from cyanobacteria to human beings.^25^, ^26^, ^27^ In recent years, a vast number of studies have documented the involvement of melatonin in cardiac protection.^28^, ^29^, ^30^, ^31^, ^32^, ^33^ Melatonin presumably enters mitochondria through oligopeptide transporters.^34^ Measurement of the subcellular distribution of melatonin showed that the concentration of melatonin in mitochondria greatly exceeds that in blood.^35^, ^36^ This evidence suggests that melatonin may specifically target mitochondria. Onphachanh and colleagues demonstrated that melatonin stimulates PINK1 expression via the MT2/Akt/NF‐κB pathway, which is important for the prevention of neuronal cell injury under high glucose conditions.^37^ Our previous study also demonstrated that melatonin alleviates cardiac remodelling and dysfunction in DCM.^38^ However, it is still unclear whether PINK1/PARKIN‐mediated mitophagy is the trigger for diabetic cardiomyopathy and, if so, whether melatonin is capable of alleviating diabetic cardiomyopathy by regulating mitophagy. To solve this problem, we generated Parkin^−/−^ mice to investigate the effects of melatonin on cardiomyocyte mitophagy under diabetic stress as well as the underlying mechanisms.

## 2 MATERIALS AND METHODS

### 2.1 Reagents

Melatonin (HY‐B0075) and 3‐methyladenine (3‐MA, HY‐19312) were purchased from Medchem Express (Monmouth Junction, NJ, USA). Chloroquine (CHQ, C6628) was purchased from Sigma (St. Louis, MO, USA). A terminal deoxynucleotidyl transferase‐mediated dUTP nick end labelling (TUNEL) assay kit was purchased from Roche Molecular Biochemicals (Mannheim, Germany). MitoTracker Red CMXRos (MTR, M7512), MitoTracker Green FM (MTG, M7514), and LysoTracker Green DND‐26 (LTG, L7526) were obtained from Molecular Probes Invitrogen (Carlsbad, CA, USA). The Mitochondria Isolation Kit (C3601, C3606) and ATP bioluminescent assay kit (S0026) were obtained from Beyotime (Shanghai, China). All adenoviruses (Ad‐sh‐Parkin, Ad‐sh‐Mst1, Ad‐Mst1, Ad‐GFP‐LC3) were constructed by Hanbio Technology Ltd. (Shanghai, China). The antibodies used were as follows: PGC‐1α (1:1000, ab54481), NRF‐1 (1:5000, ab175932), TFAM (1:2000, ab131607), Mst1 (1:10000, ab51134), Parkin (1:1000 for western blot and 1:100 for immunofluorescence, ab15954), p‐Parkin (Ser65; 1:800, ab154995), p62 (1:1000, ab91526), PINK1 (1:500, ab23707), and COX4 (1:2000, ab202554) were obtained from Abcam (Cambridge, UK). LC3 (1:1000, #12741S), β‐tubulin (1:1000, #2128S), and GAPDH (1:1000, #5174S) were purchased from Cell Signalling Technology (CST, Danvers, MA, USA). Secondary antibodies were conjugated with horseradish peroxidase (anti‐rabbit IgG, 1:5000, ab6721; Abcam) and FITC fluorescence‐conjugated secondary antibodies (AP187F, 1:100; Millipore, Billerica, MA, USA).

### 2.2 Animals and pharmaceutical intervention

The in vivo study was implemented in accordance with the NIH guidelines on the use of laboratory animals. All animal protocols were approved by the Institutional Animal Care of the Fourth Military Medical University.

Parkin‐null (Parkin^−/−^) mice (C57BL/6 background) were obtained from Jackson Laboratories (No.006582; Bar Harbor, ME, USA). Mst1‐null (Mst1^−/−^) and Mst1 transgenic (Mst1 Tg) mice (C57BL/6 background) were generated by K&D Gene Technology (Wuhan, China). After multiplying, gene‐modified mice were identified by western blot and real‐time PCR analyses. The littermates of wild‐type mice were used as controls. As previously described,^39^ eight‐week‐old mice (20‐25 g, male) were given an intraperitoneal injection of streptozotocin (50 mg/kg for 5 consecutive days) to induce the diabetes model. Blood glucose was measured 7 days after the last dose of STZ, only those with random blood glucose levels ≥16.6 mmol/L can be labelled with diabetes. After dissolving with ethanol, melatonin was diluted in distilled water at a dose of 20 mg/kg/d and then administered by oral gavage for 4 weeks.^39^

Several groups were set as follows: (a) wild type (WT, n = 32); (b) wild type + melatonin (Mel, n = 32) (c) diabetes (DM, n = 30); (d) diabetes + melatonin (DM + Mel, n = 30); (e) DM + Parkin^−/−^ (n = 24); (f) DM + Parkin^−/−^ + Mel (n = 25); (g) DM + Mst1^**−/−**^ (n = 21); (h) DM + Mst1‐Tg (n = 20); (i) DM + Mst1^**−/−**^ + Mel (n = 20); and (j) DM + Mst1‐Tg + Mel (n = 20).

### 2.3 Primary neonatal cardiomyocyte culture, transfection and treatment

The ventricles of the neonatal heart were isolated from 1‐day‐old wild‐type C57BL/6 mice and digested enzymatically as previously described.^40^, ^41^ Cardiomyocytes were adhered to dishes and grown for 48 hours and then transduced with recombinant adenoviruses harbouring the control vector (Ad‐LacZ), Parkin shRNA (Ad‐sh‐Parkin), Mst1 (Ad‐Mst1) or Mst1 shRNA (Ad‐sh‐Mst1) for 48 hours with a MOI of approximately 80‐100:1. Next, cells were cultured in 100 μmol/L melatonin for 4 hours and subjected to high‐glucose culture for 48 hours.^39^ Finally, cells were incubated for 4 hours with 3‐methyladenine (3‐MA) or chloroquine (CHQ) to inhibit autophagy when necessary.^42^

### 2.4 Fluorescence image analysis

Colocalization of mitochondria with LC3, lysosome or Parkin was used to visually assess mitophagy.^43^ Adenoviruses with GFP‐LC3 were incubated with primary cardiomyocytes for 24 hours to visualize LC3 punctae. Cells were dyed with MitoTracker Red CMXRos (MTR, 50 nmol/L) for 25 minutes at 37°C to visualize mitochondria and stained with LysoTracker Green DND‐26 (LTG, 100 nmol/L) for 60 minutes to detect lysosomes. Following staining by MTR and fixation with 4% paraformaldehyde, anti‐Parkin and FITC‐conjugated secondary antibodies were used to visualize the Parkin distribution according to previously described protocols.^39^ Images were visualized under an Olympus FV1000 laser confocal microscope and analysed using ImageJ software. Data were performed in three independent experiments.

### 2.5 Adult cardiomyocyte isolation

Mice were anaesthetized with 4% chloral hydrate and fastened in the supine position. As previously reported,^44^ the heart was removed and perfused with Ca^2+^ free Tyrode's Solution (Sigma, L6402) by Langendorff‐based methods to clear the blood. Next, continuous perfusion with Tyrode's Solution containing 0.1% collagenase II was applied to pretreat cardiac tissues that were minced into small chunks. After efficient mixing and digesting, cells were incubated in DMEM containing 10% foetal bovine serum and then filtered through a 200‐μm strainer. Following centrifugation, isolated adult cardiomyocytes were reserved for analysis.

### 2.6 Flow cytometry

Flow cytometric analysis of cellular mitochondria was performed. Cardiomyocytes were incubated with Trypsin‐EDTA for 5 minutes at 37°C and then suspended in DMEM containing 10% foetal bovine serum. Adherent cells were separated to monocells by soft pipetting. Next, cells were collected, centrifuged, resuspended, and incubated with MitoTracker Green (50 nmol/L) for 30 minutes at 37°C. After normalization with unstained cells, approximately 10 000 cells each group were analysed by measuring the mean fluorescence intensity (Beckman Coulter Cytomics FC500 Flow Cytometer, Indianapolis, IN, USA). This experiment was repeated three times.

### 2.7 Assessment of cardiomyocyte apoptosis

A TUNEL assay kit was used to evaluate the apoptosis ratio of the cardiomyocytes as described before.^39^ Data were representative of three independent experiments; random 20 fields with ×200 were assessed per group.

### 2.8 Cardiac function assessment

Four months after STZ injection, echocardiography was employed to evaluate cardiac function. First, the mice were anaesthetized through inhaling 1.5%‐2% isoflurane for examination. As previously described,^45^ The left ventricular end‐systolic diameter (LVESD) and left ventricular end‐systolic diameter (LVESD) were quantified through a M‐mode echocardiography system with a 15‐MHz linear transducer (VisualSonics Vevo 2100, Toronto, ON, Canada). Then, LVEF and LVFS were calculated using computer algorithms. Those diameters were obtained from three consecutive cardiac cycles and averaged. Data were representative of three biological repeats.

### 2.9 Transmission electron microscopy

Transmission electron microscopy (TEM) was used to observe the mitochondrial ultrastructure and typical autophagosomes engulfing a mitochondrion.^39^, ^46^ The detailed protocols were described in a previous report.^45^ The observation of TEM was repeated three times, and 12 fields with ×9900 or cells were observed per group.

### 2.10 Mitochondria and cytosol isolation

Mitochondria and cytosol were isolated using a commercial mitochondria isolation kit according to the manufacturer's instructions. First, fresh, minced heart tissues or primary collected cardiomyocytes were homogenized in Mitochondria Isolation Solution containing PMSF on ice for 15 minutes. After grinding with a glass homogenizer, centrifugation at 800 *g* was performed for 10 minutes at 4°C. The supernatant was collected in another tube and centrifuged again at 11 000 *g* for 10 minutes. The pellet was resuspended with Mitochondrial Lysate Solution to obtain mitochondrial proteins. The supernatant fraction was centrifuged at 12 000 *g* for 20 minute to obtain cytosol proteins.

### 2.11 Mitochondria functional analysis

Citrate synthase (CS) activity, ATP contents and reactive oxygen species (ROS) production were measured to estimate the mitochondrial status. CS activity was measured by a commercial assay kit (Sigma) following the manufacturer's instructions.^41^ An ATP bioluminescent assay kit (S0026; Beyotime, Shanghai, China) was used to detect the ATP level of heart tissues.^38^ After homogenizing and centrifuging at 12 000 *g* for 15 minutes at 4°C, the supernatant was mixed with the working reagent and then assessed by a microplate luminescence luminometer. As before, the contents of ROS were assessed by electron paramagnetic resonance spectroscopy according to Mellin's methods.^41^ Data were representative of three biological repeats.

### 2.12 Western blot

The detailed protocol of the western blot assay has been described previously.^38^, ^39^ Cardiac tissues or cardiomyocytes were harvested and homogenized with RIPA buffer on ice. After centrifuging at 13 000 *g* for 15 minutes and quantifying with a Bradford protein assay, protein samples were separated by SDS‐PAGE, transferred, incubated with antibodies, and scanned by a chemiluminescence system. Finally, the bands were quantified using the Image Pro Plus software (Media Cybernetics, Rockville, MD, USA). Data were representative of three independent experiments.

### 2.13 Statistical analyses

Numerical data are expressed as the mean ± standard deviation (SD) of at least three independent experiments. Significant differences between treatments were compared using an unpaired Student's *t* test, one‐way ANOVA followed by Fisher's post hoc comparison test or two‐way ANOVA with multiple post hoc comparisons. Two‐sided tests were used throughout the study, and a *P* value <0.05 was considered statistically significant. SPSS software package version 14.0 (SPSS, Chicago, IL, USA) was used for data analysis, and graphical representations were prepared by Prism software (GraphPad).

## 3 RESULTS

### 3.1 Melatonin enhances impaired mitochondria elimination in diabetic hearts

**Figure 1:**
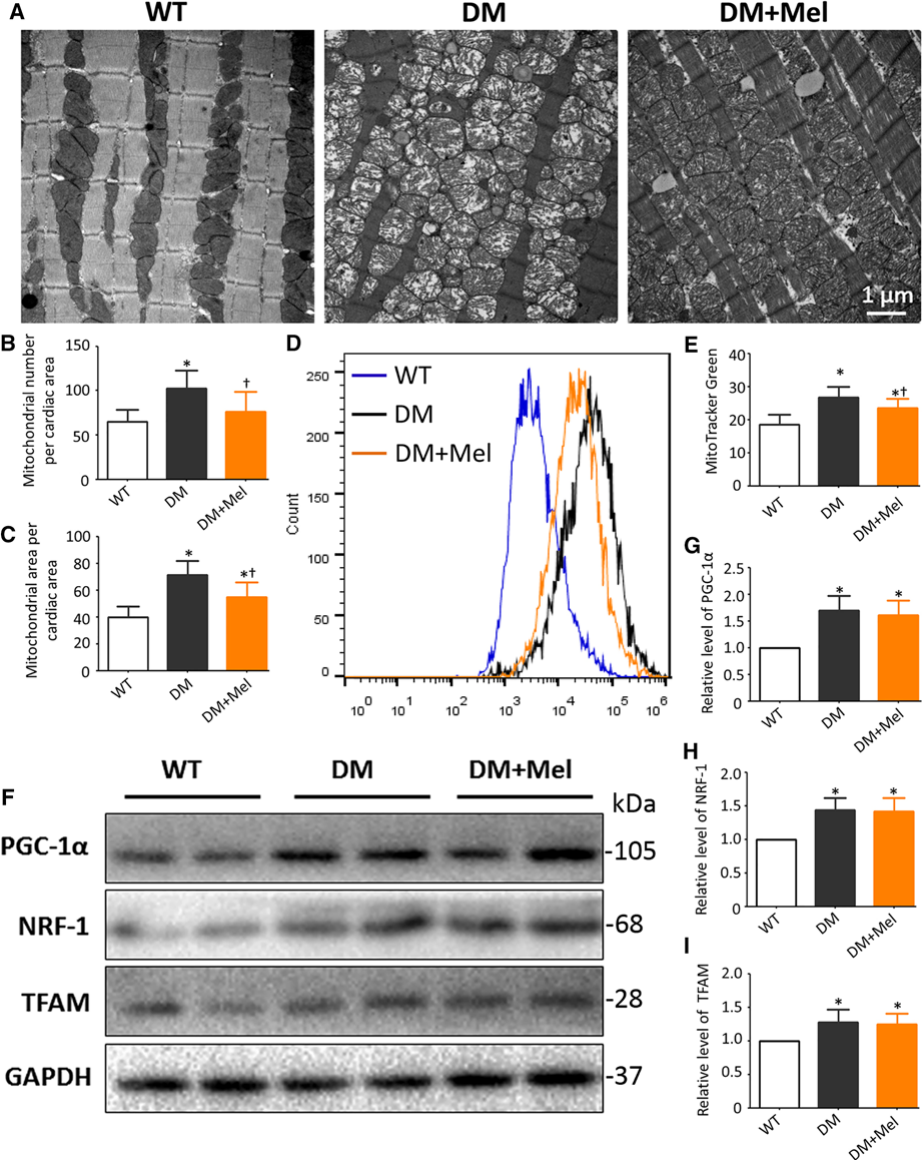
Melatonin enhances impaired mitochondria elimination in diabetic cardiomyopathy mice A, Representative TEM images of the longitudinal left ventricular wall. B, Mitochondrial number obtained by manual counting per photograph (Magnification, ×9900) per group. C, The percentage of mitochondrial areas per image (Magnification, ×9900). The columns and error bars represent the means and standard deviations (SD) (n = 12) *P < 0.05 vs WT and ^†^ *P* < 0.05 vs DM. D, Representative flow cytometric curves of the fluorescence intensity (MitoTracker Green) in adult cardiomyocytes. E, Mean fluorescence intensity (MitoTracker Green) of flow cytometric analysis. F, Protein expression with representative gel blots of PGC‐1α, NRF‐1, TFAM, and GAPDH (loading control). G, Relative levels of PGC‐1α. H, Relative levels of NRF‐1. I, Relative levels of TFAM. The columns and error bars represent the means and standard deviation (SD) (n = 4) *P < 0.05 vs WT and ^†^ *P* < 0.05 vs DM

In agreement with a previous report,^39^ electron microscopy revealed that the mitochondrial number and volume density per equivalent fields were obviously increased in diabetic mouse hearts compared with non‐diabetic controls. Melatonin markedly reduced the amount and cross section area of mitochondria in diabetic hearts (Figure 1A‐C). Parallel experiments with flow cytometric analysis demonstrated that melatonin decreased the intensity of MitoTracker Green (a mitochondrion‐specific dye) in adult cardiomyocytes subjected to diabetic insult (Figure 1D‐E). These results indicate that melatonin may reduce the number of mitochondria in the diabetic mouse heart. Subsequently, protein expression analysis was used to investigate whether inhibited mitochondrial biogenesis was responsible for the reduced mitochondrial number. Intriguingly, melatonin treatment did not significantly alter the expression of PGC‐1α, NRF‐1 and TFAM in diabetic mice (Figure 1F‐I). These results suggest that melatonin may enhance the clearance of cardiac mitochondria under diabetic conditions.

### 3.2 Melatonin up‐regulates mitophagy in diabetic cardiomyopathy

**Figure 2:**
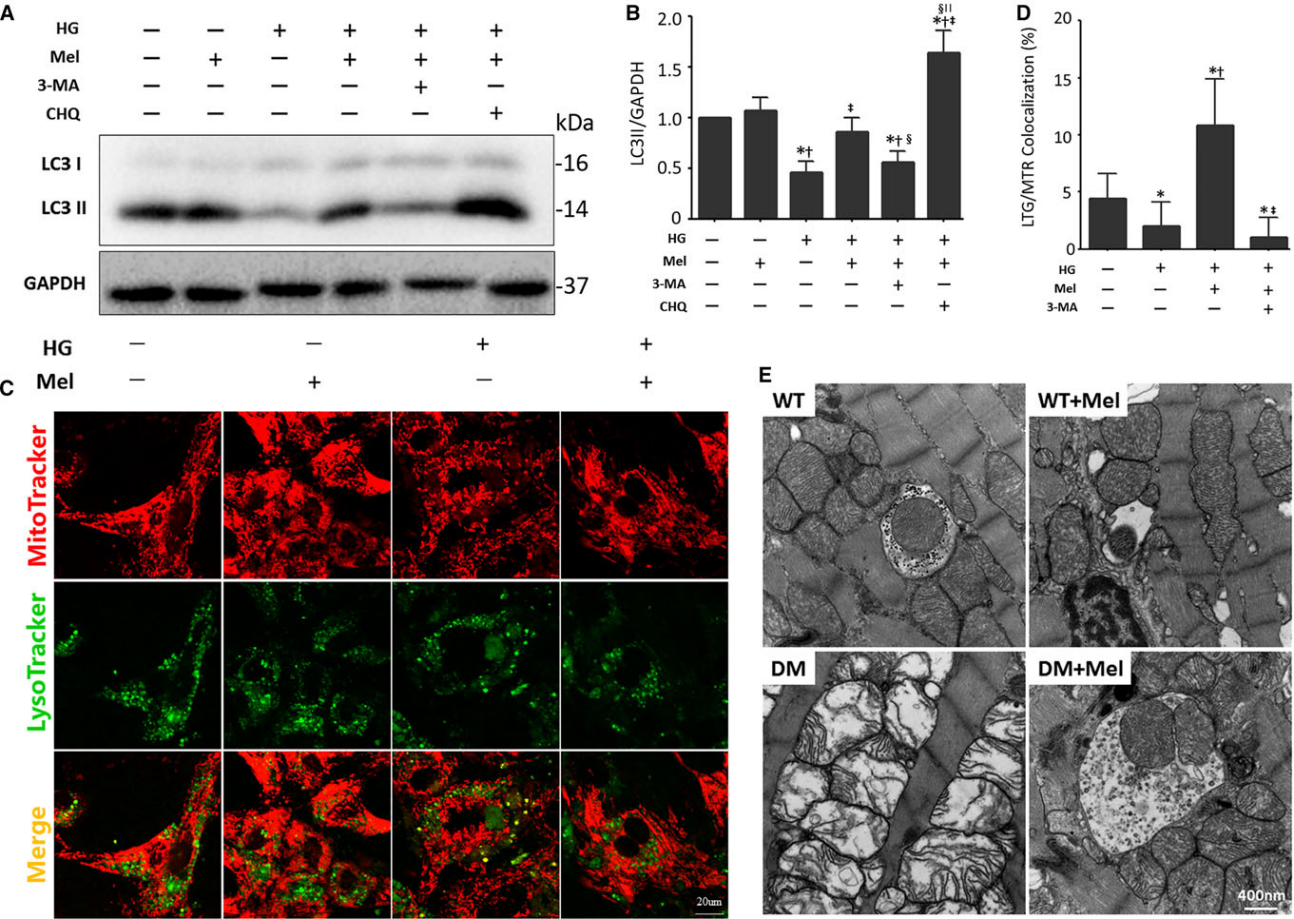
Melatonin regulation of mitophagy activity in the diabetic myocardium A, Protein expression with representative gel blots of LC3 I/II, GAPDH (loading control). B, Relative level of LC3 II. The columns and error bars represent the means and standard deviations (SD) (n = 4) *P < 0.05 vs Con; ^†^ *P* < 0.05 vs Mel; ^‡^ *P* < 0.05 vs HG; ^§^ *P* < 0.05 vs HG + Mel; ^||^ *P* < 0.05 vs HG + Mel + 3‐MA. C, Representative colocalization images of lysosomes (LysoTracker Green) and mitochondria (MitoTracker Red) (Scale bar: 20 μm). D, Quantitative analysis of LysoTracker/MitoTracker colocalization (percentage of whole cell). The columns and error bars represent the means and standard deviation (SD) (n = 30 cells) *P < 0.05 vs Con; ^†^ *P* < 0.05 vs HG; ^‡^ *P* < 0.05 vs HG + Mel. E, Representative typical autophagosome engulfing an impaired mitochondrion (Scale bars: 400 nm) in cardiac tissue

We further investigated whether melatonin mediates mitophagy activity to purge damaged mitochondria from the diabetic myocardium. First, we measured LC3 II expression with 3‐MA and HCQ as indicators of autophagy flux. The increased LC3 II expression exerted by melatonin was offset by 3‐MA, but grossly amplified by HCQ, indicating that melatonin prompted autophagic flux in high‐glucose‐treated cardiomyocytes (Figure 2A‐B). Next, primary cultured neonatal cardiomyocytes were stained with MitoTracker Red and LysoTracker Green, labelling mitochondria and lysosomes, respectively, to determine the effect of melatonin on mitophagy. Cardiomyocytes subjected to high‐glucose insults had lower colocalization of mitochondria and lysosomes than normal‐glucose‐treated cardiomyocytes. Pharmacological treatment with melatonin markedly improved the colocalization of mitochondria and lysosomes when cardiomyocytes were subjected to high‐glucose insult. The upregulated colocalization was notably counteracted by 3‐MA, which is an inhibitor of autophagosome formation (Figure 2C‐D). These results indicate that melatonin increases the initiation of mitophagy to degrade dysfunctional mitochondria. In parallel, typical autophagosomes engulfing mitochondria were rarely seen in diabetic heart sections under electron microscopy. Melatonin significantly increased the number of typical autophagosomes engulfing mitochondria (Figure 2E). These data indicate that melatonin rescues the impaired mitophagy activity of diabetic cardiomyopathy.

**Figure 3:**
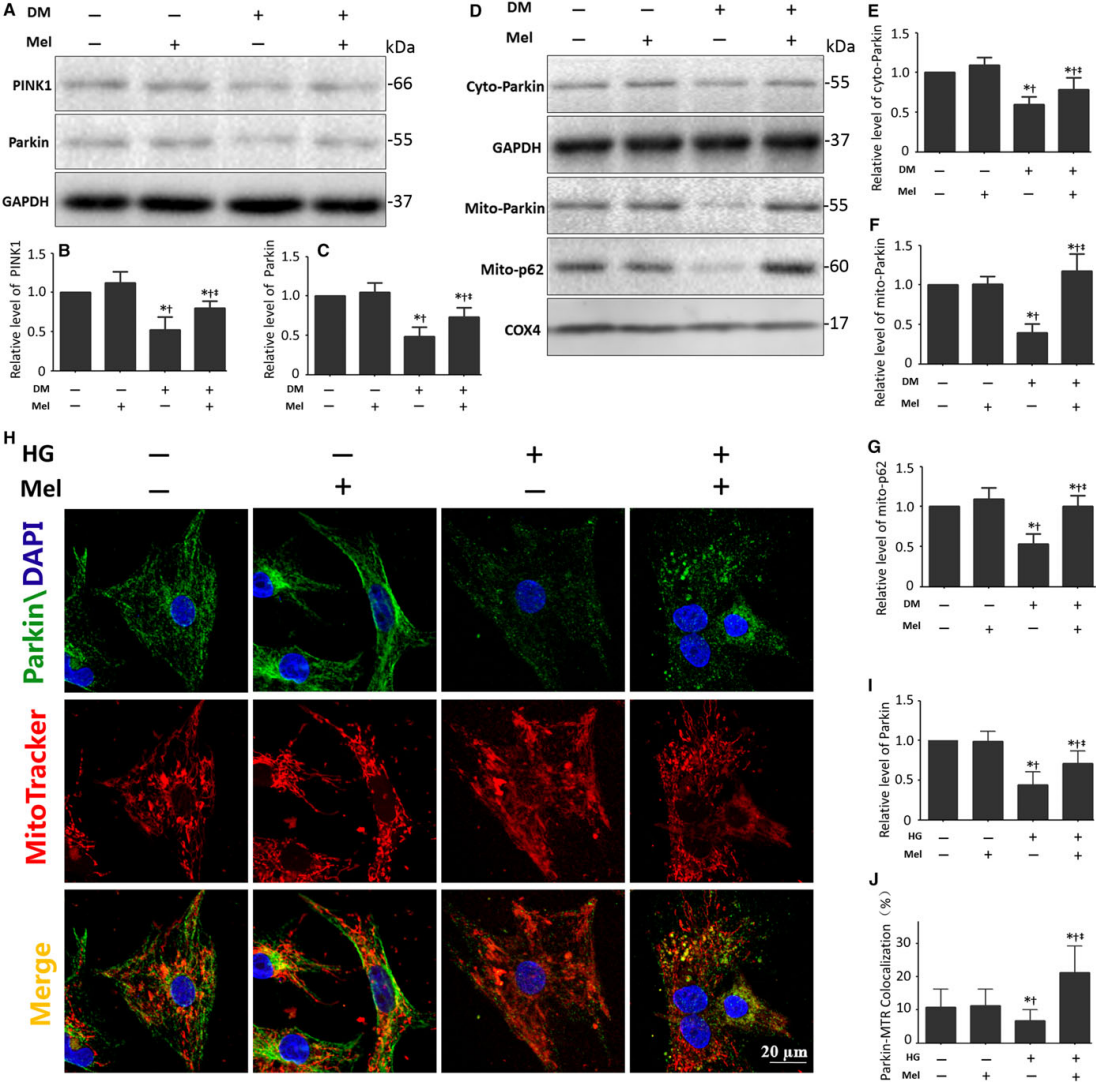
Melatonin promotes Parkin‐mediated mitophagy in diabetic cardiomyopathy A, Protein expression with representative gel blots of PINK1, Parkin, GAPDH (loading control). B, Relative levels of PINK1. C, Relative levels of Parkin. D, Protein expression with representative gel blots of Cyto‐Parkin, Mito‐Parkin, Mito‐p62, GAPDH (loading control for Cyto‐Parkin), COX4 (loading control for Mito‐Parkin, Mito‐p62). E, Relative levels of Cyto‐Parkin. F, Relative levels of Mito‐Parkin. G, Relative level of Mito‐p62. The columns and error bars represent the means and standard deviations (SD) (n = 3) *P < 0.05 vs Con; ^†^ *P* < 0.05 vs Mel; ^‡^ *P* < 0.05 vs DM. H, Representative colocalization images of Parkin (Green) and mitochondria (MitoTracker Red) (Scale bars: 20 μm). I, Relative level of Parkin in confocal images. J, Quantitative analysis of Parkin/MitoTracker Red colocalization (percentage of whole cells). The columns and error bars represent the means and standard deviation (SD) (n = 30 cells). *P < 0.05 vs Con; ^†^ *P* < 0.05 vs Mel; ^‡^ *P* < 0.05 vs HG

Cardiac PINK1 and Parkin expression was significantly depressed in diabetic mice, while melatonin administration significantly increased their expression levels (Figure 3A‐C). The results suggest that melatonin may advance mitophagy by mediating the PINK1/Parkin signalling pathway. We then isolated mitochondrial and cytosolic proteins from primary cultured cardiomyocytes. Consistently, the Parkin protein levels in mitochondria were dramatically increased by melatonin in cardiomyocytes subjected to a high glucose treatment (Figure 3D‐F). The p62 protein levels located on mitochondria were dependent on Parkin and were independent of FUNDC1, BNIP3 and BNIP3L/NIX. Thus, the elevated p62 expression in mitochondria in the presence of melatonin supports the hypothesis that melatonin enhances Parkin‐mediated mitophagy (Figure 3D and G). Furthermore, melatonin increased the immunofluorescence intensity of Parkin and colocalization of Parkin and mitochondria (Figure 3H‐J). These results are consistent with melatonin regulating Parkin‐mediated mitophagy in diabetic cardiomyopathy.

### 3.3 Parkin inhibition counteracts the effects of melatonin on mitophagy in high‐glucose‐treated cardiomyocytes

**Figure 4:**
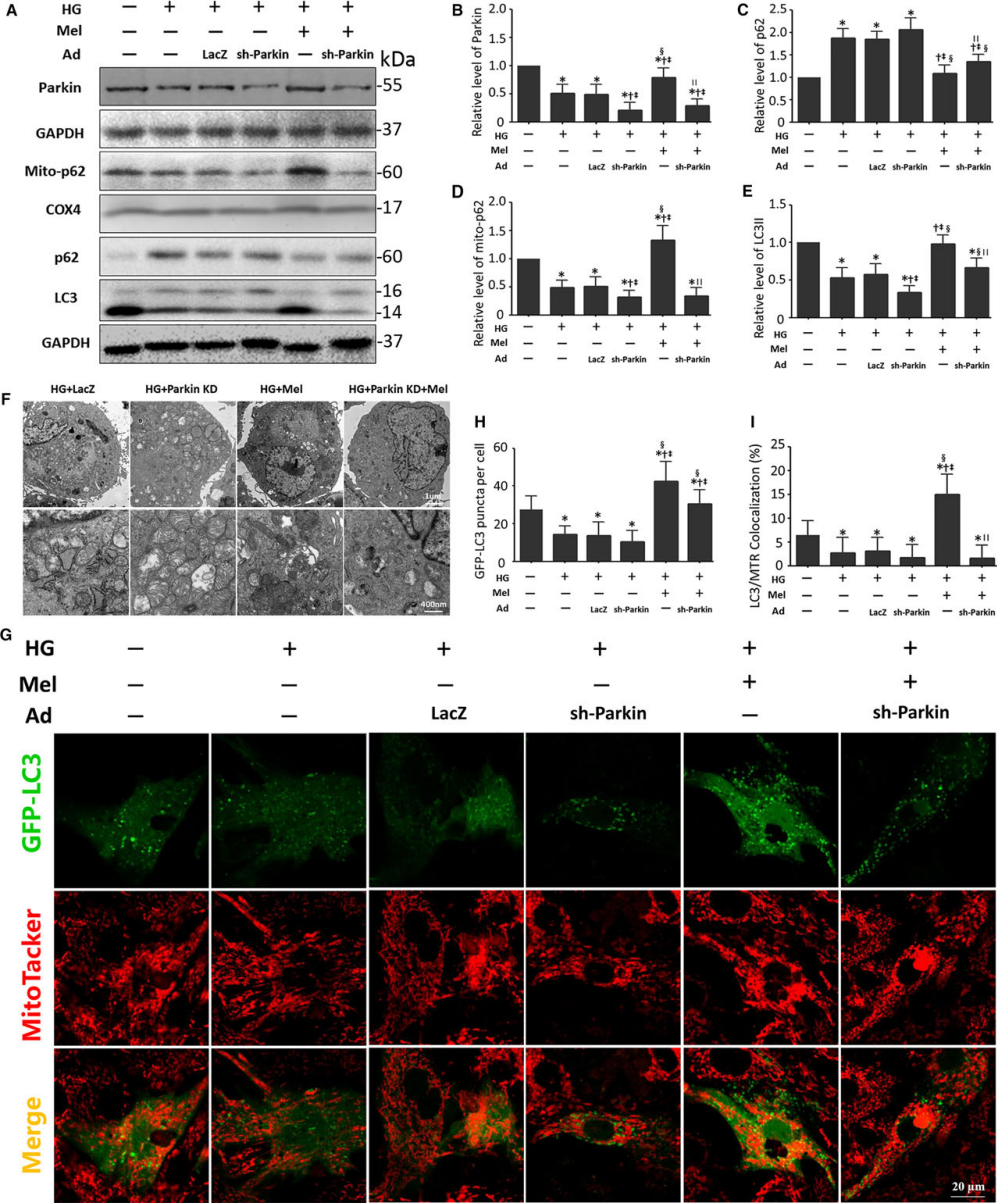
Parkin inhibition counteracts the effects of melatonin on mitophagy. A, Protein expression with representative gel blots of Parkin, Mito‐p62, COX4, p62, LC3, GAPDH (loading control for Parkin, p62, LC3). B, Relative level of Parkin. C, Relative level of p62. D, Relative level of Mito‐p62. E, Relative level of LC3 II. (n = 4). *P < 0.05 vs Con; ^†^ *P* < 0.05 vs HG; ^‡^ *P* < 0.05 vs HG + Ad‐LacZ; ^§^ *P* < 0.05 vs HG + Ad‐sh‐Parkin; and ^||^ *P* < 0.05 vs HG + Mel. F, Representative cardiomyocyte images of transmission electron microscopy (TEM) (Scale bars: upper panel 1 μm, and lower panel 400 nm). G, Representative colocalization images of GFP‐LC3 (Green) and mitochondria (MitoTracker Red) (Scale bars: 20 μm). H, Quantitative analysis of GFP‐LC3 punctae per cell. I, Percentage of cells with LC3 and mitochondria (MitoTracker Red, MTR) colocalization. The columns and error bars represent the means and standard deviations (SD) (n = 30 cells). *P < 0.05 vs Con; ^†^ *P* < 0.05 vs HG; ^‡^ *P* < 0.05 vs HG + Ad‐LacZ; ^§^ *P* < 0.05 vs HG + Ad‐sh‐Parkin; and ^||^ *P* < 0.05 vs HG + Mel

Next, we investigated whether melatonin regulation of cardiomyocyte mitophagy involved mainly through a Parkin‐dependent pathway. As expected, melatonin increased the LC3 II protein levels and decreased the p62 protein levels in cardiomyocytes under high glucose treatment. These effects were partly offset by Parkin knockdown (Figure 4A‐C and E). Additionally, Parkin inhibition counteracted the elevated p62 expression in mitochondria caused by melatonin treatment in cardiomyocytes under a high glucose treatment. These results illustrate the essential role of Parkin in melatonin‐mediated p62 recruitment and subsequent mitophagy (Figure 4A and D).

Melatonin consistently increased the number of typical autophagosomes containing damaged mitochondria in high‐glucose‐treated cardiomyocytes. These effects were obviously reversed by Parkin knockdown, as observed by electron microscopy (Figure 4F). Next, the mitophagy levels were quantified by the colocalization of GFP‐LC3 and MitoTracker Red via confocal imaging. The colocalization of GFP‐LC3 and MitoTracker Red also indicated that melatonin enhanced mitophagy to purge damaged mitochondria mostly through a Parkin‐dependent pathway (Figure 4G‐I, Figure S1).

### 3.4 Parkin deletion reverses the protective effects of melatonin on the DCM phenotype

**Figure 5:**
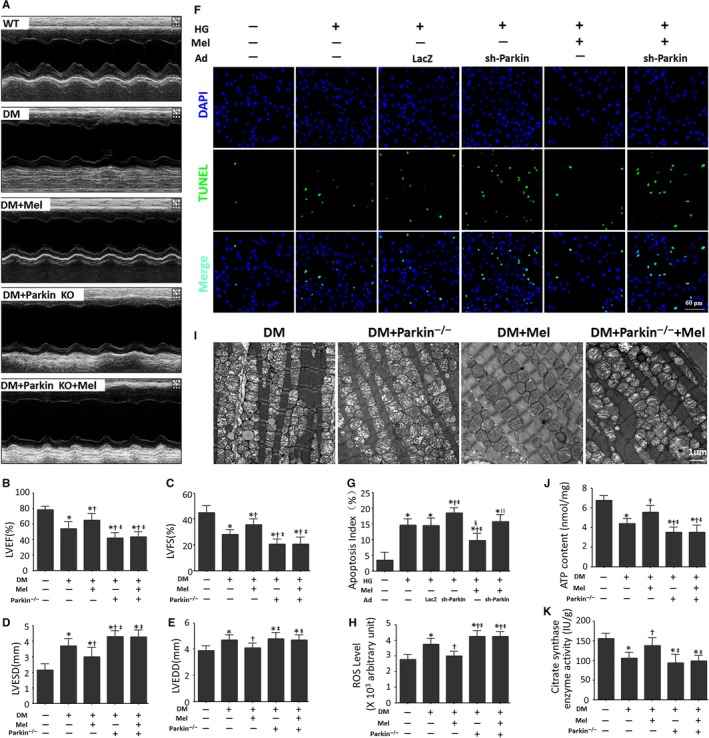
Melatonin ameliorates DCM phenotypes via Parkin. A, Representative images of echocardiography. B, Measurements of LVEF (%). C, Measurements of LVFS (%). D, Measurements of LVESD (mm). E, Measurements of LVEDD (mm). The columns and error bars represent the means and standard deviations (SD) (n = 12). *P < 0.05 vs WT; ^†^ *P* < 0.05 vs DM; and ^‡^ *P* < 0.05 vs DM + Mel. F, Representative images of TUNEL staining (Scale bar: 40 μm). G, Quantitative analysis of the apoptotic index (percentage of TUNEL‐positive nuclei, %). The columns and error bars represent the means and standard deviations (SD) (n = 20) *P < 0.05 vs Con; ^†^ *P* < 0.05 vs HG; ^‡^ *P* < 0.05 vs HG + Ad‐LacZ; ^§^ *P* < 0.05 vs HG + Ad‐sh‐Parkin; and ^||^ *P* < 0.05 vs HG + Mel. H, Relative ROS level. I, Representative images of mitochondrial morphology (Scale bars: 1 μm). J, Myocardial ATP content. K, Citrate synthase (CS) activity in cardiac tissues. The columns and error bars represent the means and standard deviation (SD). *P < 0.05 vs WT; ^†^ *P* < 0.05 vs DM; and ^‡^ *P* < 0.05 vs DM + Mel

Clearance of damaged mitochondria in cardiomyocytes is crucial for mitochondrial quality control and cardiac function. Subsequently, we examined whether Parkin‐mediated mitophagy was essential for the protective effects of melatonin on cardiac remodelling in diabetic cardiomyopathy. Consistent with our previous results, melatonin significantly reduced LVESD and LVEDD and increased LVEF and LVFS in mice with DCM (Figure 5A‐E). These protective effects of melatonin on cardiac remodelling were partially reversed by Parkin knockout (Figure 5A‐E, Table S1), suggesting that Parkin‐mediated mitophagy contributed to the favourable effects of melatonin in mice with DCM. Furthermore, melatonin did not further reduce the apoptosis index and ROS production of high‐glucose‐treated cardiomyocytes when Parkin was knocked down (Figure 5F‐G).

Parkin knockout also counteracted the beneficial effects of melatonin on cardiac mitochondrial morphology and the bioenergetic disorder of diabetic mice. The swollen mitochondria and fragmented mitochondrial cristae in diabetic hearts were alleviated by melatonin administration and were aggravated by Parkin knockout (Figure 5I). Parkin knockout also abolished the favourable effects of melatonin on ATP production and CS activity in the diabetic myocardium (Figure 5I and K). These results indicated that melatonin improved the myocardial phenotype and mitochondrial function via Parkin‐dependent mitophagy.

### 3.5 Melatonin enhances Parkin‐mediated mitophagy through Mst1 suppression

**Figure 6:**
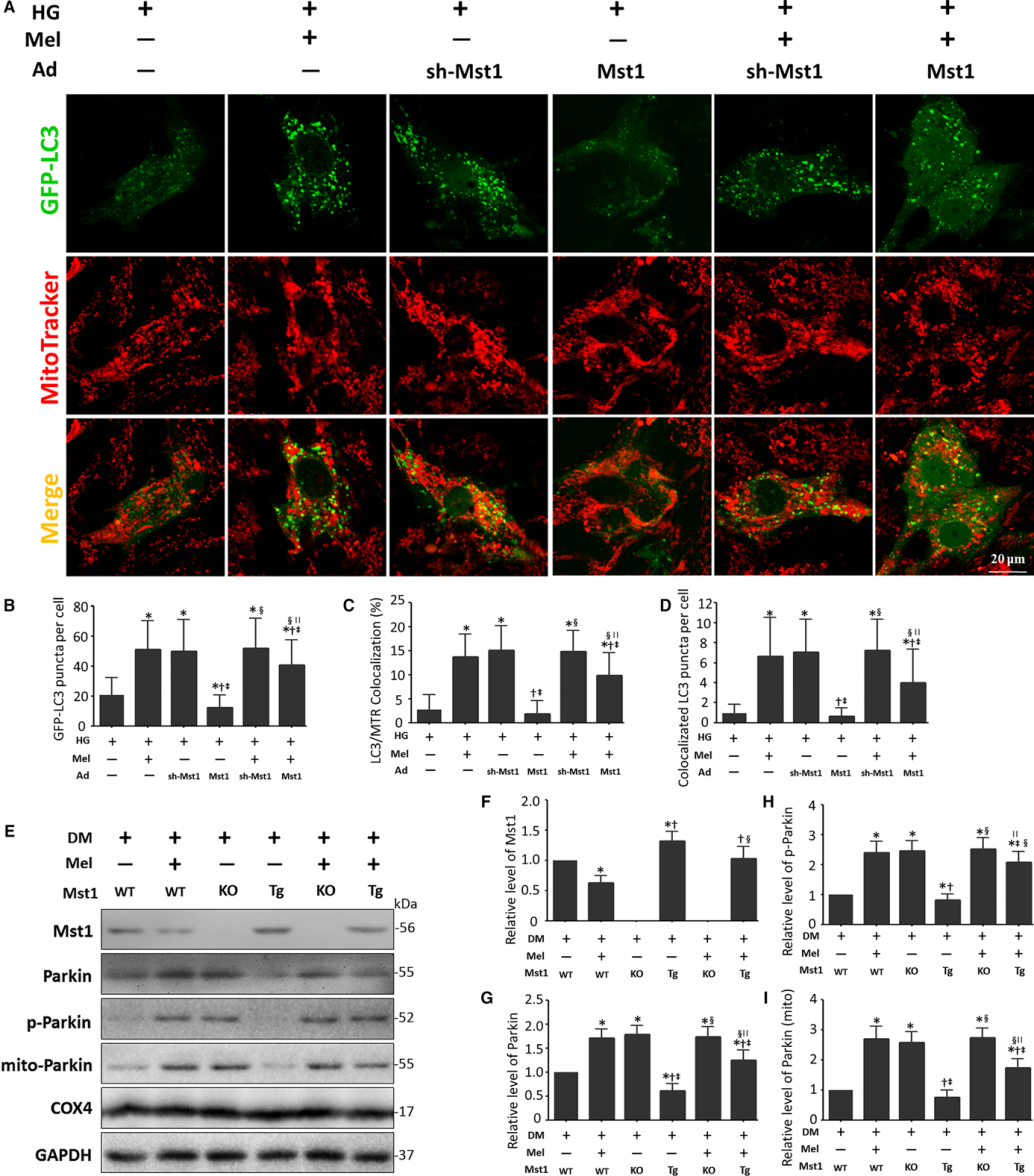
Mst1 as a negative regulator amongst melatonin‐regulated Parkin signaling. A, Representative colocalization images of GFP‐LC3 and mitochondria (MitoTracker Red) (Scale bars: 20 μm). B, Quantitative analysis of GFP‐LC3 punctae per cell. C, Percentage of cells with LC3 and mitochondria (MitoTracker Red, MTR) colocalization. D, Quantitative analysis of GFP‐LC3 puncta colocalization with mitochondria (MitoTracker Red) per cell. The columns and error bars represent the means and standard deviations (SD) (n = 30 cells). *P < 0.05 vs HG; ^†^ *P* < 0.05 vs HG + Mel; ^‡^ *P* < 0.05 vs HG + Ad‐sh‐Mst1; ^§^ *P* < 0.05 vs HG + Ad‐Mst1; and ^||^ *P* < 0.05 vs HG + Ad‐sh‐Mst1 + Mel. E, Representative gel blots of Mst1, Parkin, p‐Parkin (Ser65), Mito‐Parkin, COX4 (loading control for mitochondrial protein) and GAPDH (loading control for whole protein). F, Relative levels of Mst1. G, Relative levels of Parkin. H, Relative levels of p‐Parkin. I, Relative levels of Mito‐Parkin. The columns and error bars represent the means and standard deviations (SD) (n = 4). *P < 0.05 vs DM; ^†^ *P* < .05 vs DM + Mel; ^‡^ *P* < 0.05 vs DM + Mst1^−/−^; ^§^ *P* < 0.05 vs DM + Mst1 Tg; and ^||^ *P* < 0.05 vs DM + Mst1^−/−^ + Mel

Our previous results demonstrated that melatonin inhibited Mst1 phosphorylation and eased diabetic insults in cardiomyocytes. Thus, we further analysed whether Mst1 plays a pivotal role in melatonin‐mediated mitophagy. First, we investigated the effects of Mst1 on mitophagy in primary cultured cardiomyocytes subjected to high glucose. Similar to melatonin treatment, Mst1 knockdown increased the colocalization of GFP‐LC3 and mitochondria. Conversely, Mst1 overexpression markedly reduced the colocalization of GFP‐LC3 and mitochondria. Furthermore, melatonin significantly reversed the decreased colocalization of GFP‐LC3 and mitochondria exerted by Mst1 expression in high‐glucose‐treated cardiomyocytes (Figure 6A‐D).

We also investigated whether melatonin enhanced Parkin‐mediated mitophagy through Mst1 inhibition in mice with DCM. Mst1 knockout abolished the rise in expression of Parkin exerted by melatonin in diabetic mice hearts (Figure 6E‐G). Additionally, the increased mitochondrial Parkin and phosphorylated Parkin (Ser65) expression induced by melatonin was also abolished by Mst1 knockout (Figure 6H and I).

Taken together, these findings indicate that melatonin inhibits Mst1 phosphorylation, thus enhancing Parkin‐mediated mitophagy to eliminate dysfunctional mitochondria which contributes to mitochondrial quality control.

## 4 DISCUSSION

The prevalence of diabetes continues to increase the international health burden.^1^, ^47^ Diabetic cardiomyopathy affects a significant percentage of diabetic patients and greatly contributes to mortality.^2^, ^3^, ^48^ Previously, we showed that mitochondrial injury plays a primary role in the development of diabetic cardiomyopathy. The mitochondrial ATP content, diabetic myocardium citrate synthase activity, and activities of complexes I/II/III/IV/V in isolated mitochondria were significantly reduced in the diabetic and high‐glucose‐treated groups.^38^, ^39^ Recent studies also indicated that dysfunctional mitochondria accumulation can be detrimental to the function of cardiomyocytes, necessitating removal of damaged organelles for cardiomyocyte survival.

Herein, we observed accumulation of mitochondria with misarranged cristae. Melatonin pretreatment alleviated dysfunctional mitochondria accumulation. This phenomenon may be caused by decreased mitochondrial biogenesis or increased mitochondria elimination. Western blot analysis of biogenesis related proteins, including PGC‐1α, NRF‐1 and TFAM,^6^ indicated that melatonin did not affect the biogenesis of mitochondria. These results suggested that mitochondria elimination may have been responsible for the effects of melatonin on mitochondrial quality control.

In the past, it was believed that mitochondria degradation through autophagy was of low specificity. In recent years, it has become clear that mitophagy is capable of selectively removing dysfunctional mitochondria to maintain mitochondrial quality and homoeostasis.^7^, ^20^ However, the involvement of mitophagy in the pathogenesis of diabetic cardiomyopathy is far from clear.^13^ Here, we investigated the effects of melatonin on mitophagy in mice subjected to a diabetic insult. Typical autophagosomes engulfing mitochondria were found in non‐diabetic mouse hearts by using transmission electron microscopy,^46^ but that were rarely observed in the myocardia of diabetic mice. Additionally, large numbers of cristae‐disorganized mitochondria accumulated in the diabetic myocardium, suggesting that clearance of damaged mitochondria was impaired. Interestingly, melatonin elevated the number of autophagosomes engulfing cristae‐disorganized mitochondria in the diabetic myocardium. These results suggested that melatonin may up‐regulate mitophagy in the diabetic heart. This hypothesis was further confirmed by western blot analysis of LC3 II. Melatonin increased LC3 II expression, indicating that melatonin may participate in the initiation of mitophagy in the diabetic myocardium. Additionally, melatonin enhanced the colocalization of lysosome and mitochondria in diabetic cardiomyocytes, showing that melatonin enhances lysosome degradation of damaged mitochondria. Taken together, these results provide evidence that indicate that melatonin enhances mitophagy flux in cardiomyocytes subjected to diabetic insult.

The serine/threonine kinase PTEN‐induced putative kinase 1 (PINK1) and E3 ubiquitin ligase Parkin play indispensable roles in mitophagy.^49^, ^50^ Disruption of either leads to the failure of selective degradation of impaired mitochondria.^51^, ^52^ Loss of the mitochondrial membrane potential triggers PINK1 accumulation on the surface of mitochondria and phosphorylates mitochondrial outer membrane proteins.^49^, ^53^ This process accelerates Parkin translocation from the cytosol to the mitochondria,^49^, ^54^ and thus ubiquitinates mitochondrial outer membrane proteins to promote recruitment of an autophagosome.^55^ Other than the PINK1/Parkin pathway, BNIP3, BNIP3L and FUNDC1 are also emerging key players in the regulation of mitophagy.^13^, ^56^, ^57^ A full understanding of the regulatory mechanisms involved in cardiomyocyte mitophagy has yet to be developed, as well as their connection with the onset of diabetes.^13^ The present study demonstrated that the diabetic myocardium is associated with reduced expression of PINK1 and Parkin, suggesting that mitochondrial quality control via the PINK1/Parkin pathway is influenced. These results are consistent with a previous study, which indicated that mitophagy was reduced in diabetic hearts.^58^ This group also reported mitochondrial dysfunction and accumulation of dysfunctional mitochondria in the diabetic myocardium.

Melatonin pretreatment also increased expression of PINK1 and Parkin, as well as mitochondrial Parkin, the latter was documented by the immunofluorescence studies. These results indicate that melatonin up‐regulates Parkin‐mediated mitophagy, thus eliminating dysfunctional mitochondrial and maintaining the homoeostasis of mitochondria in the diabetic heart. The linkage of the ubiquitin‐binding protein p62 to ubiquitin on the mitochondrion and lipidated LC3II on the autophagosome provides a physical attachment point for mitophagy.^13^, ^22^, ^59^ The present study also indicates that mitochondrial p62 increases after melatonin administration. These results further demonstrate that melatonin stimulates mitophagy in the diabetic heart.

Diabetic cardiomyopathy is manifested as a declining contractility of the cardiomyocytes and the development of heart failure independent of coronary artery disease. In its most severe state, diabetic cardiomyopathy is manifested as a ventricular dilation and decreased cardiac output.^3^, ^4^, ^39^ Consistent with our previous results, diabetic mice exhibited left ventricular enlargement and a drop in LVEF. Parkin knockout abolished the beneficial effects of melatonin on left ventricular remodelling and cardiac dysfunction in diabetic mice. In Parkin knockout diabetic mice, melatonin did not alleviate mitochondrial cristae fragmentation, suggesting that melatonin may participate in the protection against mitochondrial injury through a Parkin‐dependent pathway. Furthermore, Parkin knockout also abolished the beneficial effects of mitochondrial bioenergetics in the diabetic heart exerted by melatonin.

Our previous study indicated that melatonin protects against diabetic cardiomyopathy through Mst1 inhibition.^38^ The role of mitophagy ensures the removal of dysfunctional mitochondria. The involvement of Mst1 in melatonin‐mediated mitophagy and clearance of mitochondria in the diabetic heart had not been previously determined. Mst1 overexpression decreased the colocalization of LC3 and mitochondria in cardiomyocytes subjected to a high glucose insult. Mst1 knockdown alleviated the effects of melatonin on the colocalization of LC3 and mitochondria. These results indicated that melatonin increased mitophagy through Mst1 inhibition. An in vivo study further demonstrated that melatonin failed to elevate mitochondrial Parkin expression, Parkin phosphorylation and mitochondrial p62 expression in cardiomyocytes treated with high glucose. The results further demonstrate that melatonin exerts protective effects by enhancing mitophagy through Mst1 inhibition and Parkin activation.

Collectively, the results reported herein indicate that impaired mitophagy contributes to diabetic cardiomyopathy due to reduced clearance of dysfunctional mitochondria. Enhancing mitophagy by melatonin alleviates dysfunctional mitochondria accumulation and restores mitochondrial quality control, thus improving cardiac function. The cardioprotective effects of melatonin may be partially explained by Mst1 inhibition as well as Parkin activation.

### 4.1 Limitations

Neonatal cardiomyocytes rather than adult cardiomyocytes were used to investigate possible mechanisms. Further studies should be performed to explore how melatonin inhibits Mst1 expression and phosphorylation. The protective effects of melatonin on mice with type 2 diabetes should also be elucidated in further studies.

## CONFLICT OF INTEREST

All authors declare that there is no conflict of interest.

## AUTHOR CONTRIBUTIONS

Dongdong Sun, Shanjie Wang, Russel J. Reiter, Haichang Wang, and Zhijing Zhao conceived the project, researched data, analysed data and wrote the manuscript. Xinyu Feng, Zheng Cheng, Zhenyu Xiong, Tingting Wang, Jie Lin, Mingming Zhang, and Jianqiang Hu researched and analysed the data. Yanhong Fan and Russel J. Reiter reviewed and edited the manuscript. Dongdong Sun is the guarantor of this work and, as such, had full access to all the data in the study and takes responsibility for the integrity and accuracy of the data analysis.
