# Supplementary material for: Melatonin activates Parkin translocation and rescues the impaired mitophagy activity of diabetic cardiomyopathy through Mst1 inhibition
Source: J Cell Mol Med. 2018 Jul 31;22(10):5132–44. doi: 10.1111/jcmm.13802 (PMC6156356; doi:10.1111/jcmm.13802)
Supplement: Supplementary file 1 [file JCMM-22-5132-s001.docx]

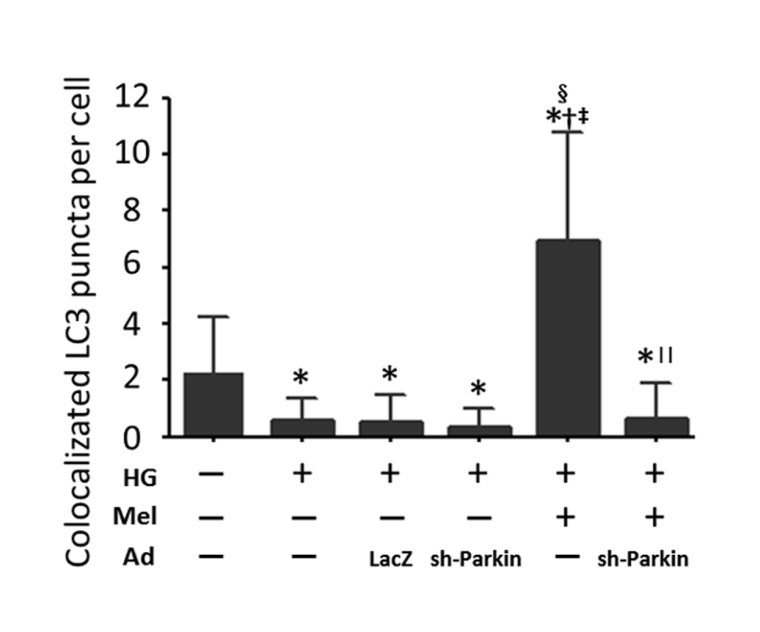


**Supplementary Figure.1 Parkin inhibition reduces LC3 puncta colocalization with mitochondria** Quantitative analysis of GFP-LC3 puncta colocalization with mitochondria (MitoTracker Red) per cell. The columns and error bars represent the means and standard deviation (SD) (n=30 cells). *P<0.05 vs. Con; † P < 0.05 vs. HG; ‡P < 0.05 vs. HG + Ad-LacZ; §P<0.05 vs. HG + Ad-sh-Parkin; and || P < 0.05 vs. HG +Mel.

**Supplementary Table. 1 Basic parameters of mice and the thickness of left ventricular posterior wall**

| **Parameters** | **WT** | **DM** | **DM+Mel** | **DM+Parkin^-/-^** | **DM+Parkin^-/-^+Mel** |
| --- | --- | --- | --- | --- | --- |
| Body weight (g) | 31±3.7 | 24±2.9* | 28±3.5^†^ | 24±3.1^‡^ | 23±3.3^‡^ |
| Heart weight (mg) | 123±21 | 101±15* | 112±19 | 93±15^‡^ | 95±14^‡^ |
| Heart rate(bpm) | 482±89 | 474±66 | 467±71 | 460±60 | 461±62 |
| LVPW;d (mm) | 0.95±0.09 | 0.86±0.11* | 0.89±0.09 | 0.8±0.12 | 0.81±0.11 |
| LVPW;s (mm) | 1.6±0.18 | 1.2±0.14* | 1.46±0.14^†^ | 1.1±0.20^‡^ | 1.16±0.18^‡^ |

LVPW =the thickness of left ventricular posterior wall, The columns and error bars represent the means and standard deviations (SD) (n= 12).

*P<0.05 vs. WT; †P < 0.05 vs. DM; and ‡P < 0.05 vs. DM + Mel.
